# Editorial: Development of *in vitro* toxicology methods using organoid systems and toxicogenomic approaches

**DOI:** 10.3389/fgene.2022.1030580

**Published:** 2022-10-03

**Authors:** Toshio Imai, Yoshitaka Hippo

**Affiliations:** ^1^ National Cancer Centre, Tokyo, Japan; ^2^ Chiba Cancer Center, Chiba, Japan

**Keywords:** organoids, toxicology, chemicals, gene modification, CRISPR/Cas

## Introduction

Organoids, also known as mini-organs, can be cultured by embedding primary cells derived from normal or cancerous tissues in Matrigel and reconstituting individual tissue stem cell niches with specific factors. Compared to conventional two-dimensional (2D) culture, their use has been expanding in numerous research areas owing to their advantages such as long-term culture under a physiological environment, high culture success rate, and faithful retention of the heterogeneity of the original tumor tissues. Toxicological evaluation and mechanistic elucidation of drugs/environmental chemicals have been conducted using immortalized 2D cell lines and/or experimental animals; however, whether the data obtained could be extrapolated to human disease is controversial in toxicological research. Moreover, treatment with chemicals at toxicologically admitted doses may induce clinical signs in animals, which may force discontinuation of the experiment. Therefore, development of an alternative method for safe assessment of such chemicals is urgently required. From this point of view, organoids have potential as a bridge between human and conventional experimental models, because they reproduce toxicologic reactions in a way similar to those of *in vivo* models, and minimize the species differences of molecular characteristics at the cellular level and Absorption, Distribution, Metabolism, Excretion properties of chemicals at the individual level, both of which would make the interpretation the data from *in vivo* models so complicated.

This Research Topic is focused on organoid-based *in vitro* models for toxicologic and pharmacological evaluation. Selected models include, but are not limited to, those of toxicogenomic approaches, non-rodent species, and genetic models for carcinogenesis.

## Organoid-based *in vitro* models for toxicologic evaluation of chemicals

Matsui and Shinozawa reviewed the potential usefulness, limitations, and future perspectives of human liver, heart, kidney, gut, and brain-derived organoids, based on predictive toxicology research and drug development. Since human organoids mimic numerous aspects of the *in vivo* physiological functions of the relevant tissues, it is likely that they could bridge the toxicological profiles between animals and humans.

Nguyen et al. conducted anthracycline analog-exposed experiments accompanied by translational proteomic analysis. They employed cardiac organoids/microtissues comprising induced pluripotent stem cell (iPSC)-derived cardiomyocytes and human heart biopsies, which provide insights into understanding their toxic mechanisms. Finally, they concluded that the *in vitro* outcomes could be linked to clinical phenomena through the integration of proteomic data.

Komiya et al. generated lung, liver, and colon-derived organoids from *gpt* delta mice, (transgenic mice commonly employed in *in vivo* genotoxicity assay) and performed comparative analysis between *in vivo* and *in vitro* treatment of the environmental chemicals. This approach enables the avoidance of chemical treatment at maximum tolerated doses *in vivo* and the detection of the intra-organoid metabolic activation capacity of the chemicals, which has not been feasible using other *in vitro* genotoxicity assay systems.

Naruse et al. confirmed the carcinogenic potential of a known carcinogen by administering it to murine mammary organoids *in vitro* and their subsequent injection to nude mice. The developed tumors in mice showed genetic characteristics distinct from those induced by oral administration of the chemical, suggesting that the organoid system could be applied to the clarification of a novel mode-of-action in chemical carcinogenesis.

These articles underscore the advantages of organoids in minimizing the gap in drug/chemical responses among species and also in the elucidation of the mode of action of toxicologic effects of drugs and environmental factors, especially by integration into toxicogenomic approaches. In addition, they illustrate future perspectives such as a co-culturing system of organoids with endothelial cells, immune cells, and other mesenchymal cells in relevant tissues/organs that would likely improve the accuracy of the toxicological assessment of each drug/chemical.

## Organoid-based disease models for pharmacology/toxicology research

The current status and future perspectives of the use of patient-derived organoids (PDOs) in cancer and toxicology research are outlined by Sekine. The advantage of PDOs include the ease of *in vitro* genetic manipulation, the availability of normal (non-cancerous) cells as well as iPSC-derived epithelial cells, and the ability to generate patient-derived xenografts. Reproducing a small number of mutations by genome editing in normal PDOs and subsequently using them for carcinogenesis and toxicology research may deepen our understanding of carcinogenesis.

A review by Kopper et al. outlined the utility of canine models and their resultant intestinal organoids in the analysis of inflammatory bowel disease (IBD). Although genetically modified mice have commonly been used as disease models for IBD, it should be noted that they do not always faithfully reproduce the pathogenesis and pathophysiology of the disease as observed in humans. In contrast, canine and human IBD share common clinical and molecular features. Based on their review, they discuss the value of gut organoids derived from canine IBD model, and how they can heighten understanding of the interplay among dietary interventions, gut microbiota, and intestinal inflammation.

## Organoid-based hybrid genetic models for carcinogenesis

A review by Maru and Hippo reviewed two genetically engineered models of endometrial cancer. One of these models consisted of *in vivo* mouse models reflecting gene mutations detectable in human endometrial cancers. Recently developed *Cre* transgenic mice with a highly specific gene recombination in the endometrial cell lineage warrant accurate *in vivo* modeling of cancers. The second model consisted of a hybrid model of lentivirus-based *in vitro* gene transduction of endometrial organoids, followed by subcutaneous transplantation into immunodeficient mice. In this modeling system, a certain combination of gene alterations was found to induce carcinosarcoma. The other combination did not develop tumors, unlike the previous study using genetically engineered mice of the identical genotype, suggesting that the functional cooperation of gene mutations with the microenvironment may play a critical role in carcinogenesis.

A review by Takeda outlined a method to validate the carcinogenicity of colorectal cancer driver mutations by taking advantage of organoids, which enable an accurate evaluation based on their genetic homogeneity. Genomic analysis of human colorectal cancers and transposon-based *in vivo* genetic screens in mice allow the identification of many candidate genes that potentially participate in colorectal multistep carcinogenesis, which have to be further validated by functional assay. Unfortunately, it was difficult for standard *Apc* inactivation-driven *in vivo* tumorigenesis models to analyze later stages of carcinogenesis, such as invasion and metastasis, because of early death due to rapid development of multiple benign tumors. On the other hand, transplantation of organoids genetically modified by CRISPR/Cas into mice resulted in the development of a single tumor, which provides a suitable period of time to fully analyze these processes.

## Conclusion

One of the greatest advantages of organoids compared to other culturing conditions is that, they can be generated from tissue samples from both healthy and diseased states. In addition, they can be established from not only mouse and human samples, but also other species, from companion animals to fish to name a few. This enables us to conduct toxicological research of environmental pollutants. Differences in toxicologic reactions to drugs/environmental chemicals and disease process among various species can also be evaluated at the cellular level. Studies on human organoids derived from iPSC and cancer tissues has remarkably increased in number recent years, and further attempts for establishment and utilization of organoids are warranted from non-cancerous human tissue and tissues from other species.

